# Coding-Complete Genome Sequence of Yada Yada Virus, a Novel Alphavirus Detected in Australian Mosquitoes

**DOI:** 10.1128/MRA.01476-19

**Published:** 2020-01-09

**Authors:** Jana Batovska, Jan P. Buchmann, Edward C. Holmes, Stacey E. Lynch

**Affiliations:** a Agriculture Victoria Research, AgriBio Centre for AgriBioscience, Bundoora, Victoria, Australia; b School of Applied Systems Biology, La Trobe University, Bundoora, Victoria, Australia; c Marie Bashir Institute for Infectious Diseases and Biosecurity, Charles Perkins Centre, School of Life and Environmental Sciences and Sydney Medical School, The University of Sydney, Sydney, New South Wales, Australia; DOE Joint Genome Institute

## Abstract

Here, we report the detection of a novel alphavirus in Australian mosquitoes, provisionally named Yada Yada virus (YYV). Phylogenetic analysis indicated that YYV belongs to the mosquito-specific alphavirus complex. The assembled genome is 11,612 nucleotides in length and encodes two open reading frames.

## ANNOUNCEMENT

Alphaviruses (genus *Alphavirus*, family *Togaviridae*) are small (10- to 12-kb) single-stranded positive-sense RNA viruses and include species important to human and animal health, such as *Chikungunya virus* and *Eastern equine encephalitis virus* (1). While these viruses are transmitted primarily by mosquitoes and pathogenic in their vertebrate hosts, there is a small complex of recently discovered alphaviruses that replicate only in mosquito cells (2–5). Here, we report the detection of an alphavirus belonging to this host-restricted complex in the Asia-Pacific region and provide the genome sequence for the novel virus, named Yada Yada virus (YYV).

Virus detection was performed using mosquitoes trapped as part of the Victorian Arbovirus Disease Control Program (6). Encephalitis virus surveillance (EVS) traps (7) were set up overnight each week in three locations in Victoria, Australia, for a total of 7 weeks in late 2016, resulting in 21 trap collections. Traps were sorted into 86 pools of up to 1,000 mosquitoes, which were homogenized in buffer AVL (Qiagen) and centrifuged. RNA was extracted from the supernatant with the QIAamp viral RNA minikit (Qiagen) and used for library preparation, which was performed using the Ovation universal transcriptome sequencing (RNA-Seq) system (NuGEN) with a customized mosquito rRNA depletion (8). The libraries were then treated with free adapter blocking reagent (Illumina) and sequenced on a HiSeq 3000 platform (Illumina) using 2 × 150 bp reads. A total of 909,467,304 paired reads were generated (mean, 10,575,201 per pool; range, 7,971,017 to 16,414,900).

Trinity v2.4.0 (9) was used to trim, normalize, and assemble the reads into contigs, which were taxonomically classified using DIAMOND BLASTx v0.9.22.123 (10) with the NCBI nonredundant (nr) database (acquired 2 September 2019) and an E value cutoff of 10^−5^. Reads were mapped to assembled contigs using BWA-MEM v0.7.17 r1188 (11). All analyses were performed using default parameters unless stated otherwise. Three of the 21 traps tested contained contigs that had the strongest BLASTx match to the mosquito-specific Eilat alphavirus (EILV). All three traps were collected in November 2016 in Mildura (latitude, 34.249617, longitude, 142.218261). The longest contig was 11,612 nucleotides (nt), with 21-fold average coverage depth and 75.7% amino acid identity to EILV. This contig represents the coding-complete YYV genome, with two open reading frames (ORFs), a 33-nt 3′ leader, a 470-nt 5′ trailer, and 53.4% G+C content. The two ORFs correspond to the structural (1,247 amino acids) and nonstructural (2,437 amino acids) proteins. Translation of the genome sequence was performed using the ExPASy Translate tool (12).

**FIG 1:**
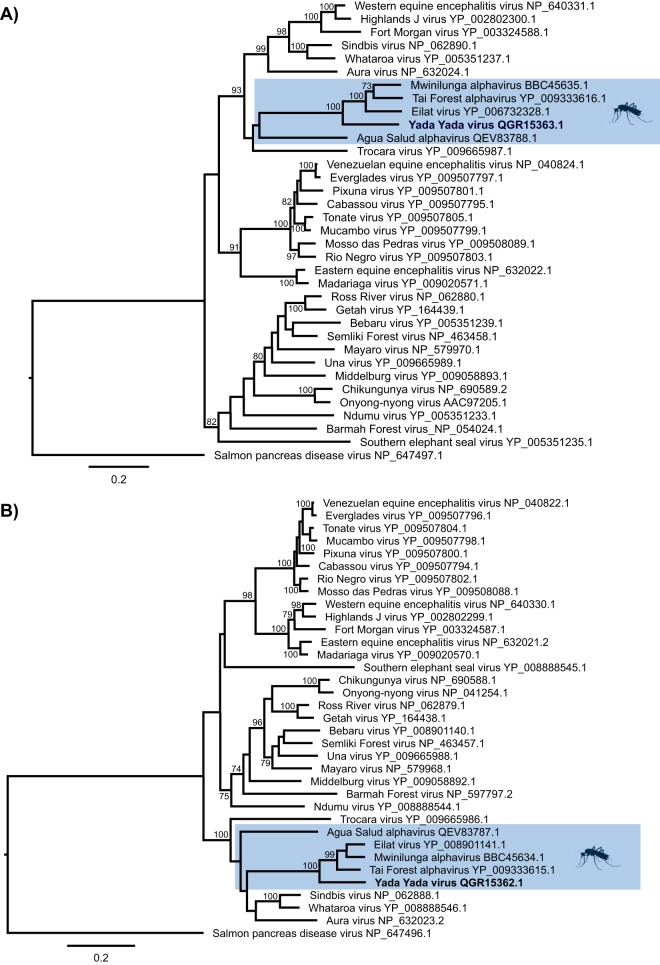
Phylogenetic relationships of YYV and other alphaviruses based on alignments of structural proteins (850 amino acids) (A) and nonstructural proteins (1,287 amino acids) (B). Maximum likelihood trees were estimated using the LG plus gamma model of amino acid substitution in PhyML, with 1,000 bootstrap replicates, and rooted using salmon pancreas disease virus. Bootstrap values greater than 70% are shown beside the branches, and the GenBank accession numbers are shown with the virus names. The mosquito-specific complex is highlighted in blue with YYV in bold.

Phylogenetic analysis was performed by the creation of alignments of YYV and the structural and nonstructural protein sequences of other alphaviruses using MAFFT v7.429 (13), the removal of ambiguously aligned residues with TrimAl v1.4.1 (14), and maximum likelihood inference using PhyML v3.1 (15) employing the Le-Gascuel (LG) plus gamma distribution model of amino acid substitution and 1,000 bootstrap replicates. The resultant trees were then viewed in FigTree v1.4.4 (16). In both the structural (Fig. 1A) and nonstructural (Fig. 1B) protein trees, YYV was placed in the mosquito-specific alphavirus complex, suggesting that it might also have a restricted host range.

To investigate the vector of YYV, the assembled contigs were compared to a cytochrome oxidase I (COI) database of Australian mosquito species (17) using BLASTn v2.9.0+ (18) with an E value cutoff of 10^−5^, and the results were filtered for alignments >200 bp in length and matches of >95% identity. Only two mosquito species were present in all three traps, Anopheles annulipes and Culex australicus/Culex globocoxitus (these two *Culex* species are indistinguishable using COI), supporting previous studies that have detected mosquito-specific alphaviruses from only *Anopheles* and *Culex* species (2–5). Due to the homogenization of the traps, the YYV vector species cannot be definitively determined. However, read mapping showed that the abundance of *A. annulipes* was associated with YYV genome coverage, whereas the abundance of *C. australicus/C. globocoxitus* was not (data not shown).

The discovery of YYV expands the diversity and geographic range of the mosquito-specific alphavirus complex and in doing so will help reveal the virus origin and evolution of host switching (19). In addition, it is noteworthy that mosquito-specific viruses that are closely related to pathogenic vertebrate viruses have potential applications in vaccine development and as biocontrol agents (20).

### Data availability.

The YYV genome sequence has been deposited in GenBank under the accession number MN733821. The sequencing reads are available in the SRA database via BioProject accession number PRJNA594295.
