# Correction for Batovska et al., “Coding-Complete Genome Sequence of Yada Yada Virus, a Novel Alphavirus Detected in Australian Mosquitoes”

**DOI:** 10.1128/MRA.00103-20

**Published:** 2020-03-05

**Authors:** Jana Batovska, Jan P. Buchmann, Edward C. Holmes, Stacey E. Lynch

**Affiliations:** a Agriculture Victoria Research, AgriBio Centre for AgriBioscience, Bundoora, Victoria, Australia; b School of Applied Systems Biology, La Trobe University, Bundoora, Victoria, Australia; c Marie Bashir Institute for Infectious Diseases and Biosecurity, Charles Perkins Centre, School of Life and Environmental Sciences and Sydney Medical School, The University of Sydney, Sydney, New South Wales, Australia

## AUTHOR CORRECTION

Volume 9, no. 2, e01476-19, 2020, https://doi.org/10.1128/MRA.01476-19. After publication, the authors were made aware of a short sequence on GenBank (JQ749729.1) with high similarity to Yada Yada virus (YYV). The 331-bp sequence is part of the nonstructural polyprotein (NSP4) and shares 97% nucleotide identity and 99.1% amino acid identity with YYV. This sequence was detected in an *Aedes notoscriptus* mosquito collected in North Melbourne, Australia, in 2011. The similarity of this sequence to YYV suggests that the virus from which it originated is part of the mosquito-specific alphavirus complex, which consists of viruses that have been detected only in *Anopheles* and *Culex* mosquitoes.

